# The Relationship Between Aggregation and Deformability of Red Blood Cells in Health and Disease

**DOI:** 10.3389/fphys.2020.00288

**Published:** 2020-04-15

**Authors:** Dan Lazari, Joames Kauffimann Freitas Leal, Roland Brock, Giel Bosman

**Affiliations:** Department of Biochemistry, Radboud Institute for Molecular Life Sciences, Radboud University Medical Center, Nijmegen, Netherlands

**Keywords:** aggregation, aging, deformability, membrane, red blood cell

## Abstract

The molecular organization of the membrane of the red blood cell controls cell morphology and function and is thereby a main determinant of red blood cell homeostasis in the circulation. The role of membrane organization is prominently reflected in red blood cell deformation and aggregation. However, there is little knowledge on whether they are controlled by the same membrane property and if so, to what extent. To address the potential interdependence of these two parameters, we measured deformation and aggregation in a variety of physiological as well as pathological conditions. As a first step, we correlated a number of deformability and aggregation parameters in red blood cells from healthy donors, which we obtained in the course of our studies on red blood cell homeostasis in health and disease. This analysis yielded some statistically significant correlations. Also, we found that most of these correlations were absent in misshapen red blood cells that have an inborn defect in the interaction between the membrane and the cytoskeleton. The observations suggest that deformability and aggregation share at least one common, membrane-related molecular mechanism. Together with data obtained after treatment with various agents known to affect membrane organization *in vitro*, our findings suggest that a phosphorylation-controlled interaction between the cytoskeleton and the integral membrane protein band 3 is part of the membrane-centered mechanism that plays a role in deformability as well as aggregation.

## RBC Aging and Disease

Tissue oxygenation depends on hemoglobin as much as on red blood cell (RBC) characteristics such as metabolism, communication with the immune system, deformability and aggregation behavior. Many if not all of these processes depend on the organization of the RBC membrane that enables the complex, dynamic interactions between the cell membrane and various intracellular and extracellular molecules. Studies of inborn errors of metabolism and membrane protein composition, in combination with the structural and functional changes that occur during RBC aging, have led to a membrane-centered molecular understanding of red blood cell function and survival, and of the role of membrane molecules in cell morphology and membrane organization. Modification of the integral membrane protein band 3 plays a central role in the weakening of the cytoskeleton/membrane interaction at the ankyrin complex, which underlies the generation of microvesicles (Bosman et al., 2010, 2012; Lutz and Bogdanova, 2013). Vesiculation results in the loss of approximately 20% of the hemoglobin, a decrease of 30% in MCV and a concomitant increase in 15% in MCHC (Willekens et al., 1997). The associated changes in cell density and volume enable a clear separation of RBCs according to cell age (Bosch et al., 1994; Willekens et al., 1997, 2008). The accumulation of band 3-derived neoantigens and phosphatidylserine on microvesicles induces their fast disappearance from the circulation, prevents inflammation and thrombosis. Overall, shedding of defective components through vesiculation prevents untimely removal of otherwise functional RBCs (Willekens et al., 2008; Bosman et al., 2012; Alaarg et al., 2013; Freitas Leal et al., 2017, 2018). Nevertheless, old and pathological RBCs are more prone to display removal signals or break down when exposed to osmotic or mechanical stress (Bosman et al., 2011; Ghashghaeinia et al., 2012).

## Deformability

In the course of our investigations on the functional consequences of aging-associated or pathology-related changes in membrane organization, we noticed multiple changes in deformability and aggregation (Cluitmans et al., 2012, 2014, 2015, 2016; Freitas Leal et al., 2019). Deformability is a critical determinant of RBC function, because of the extensive change in cell shape required for efficient passage through the capillaries and the spleen. Indeed, physiological aging of RBCs *in vivo* is associated with a pronounced decrease in their deformability (Bosch et al., 1994). Also, many RBC-centered pathologies such as deficiencies in metabolic enzymes, altered hemoglobins, and mutations in membrane proteins affect the same processes that play a critical role in physiological aging, and are associated with decreased deformability, as well (Alaarg et al., 2013; Mohandas, 2017). This may be due directly to weakening of the interactions between membrane and/or cytoskeleton proteins, or to the resulting loss of membrane by vesiculation (Huisjes et al., 2018). A decrease in the capacity to deform will lead to a decrease in tissue perfusion and oxygenation, and thereby contribute to the pathophysiology.

## Aggregation

The same RBC characteristics that determine RBC deformability also play a role in their interaction with plasma proteins, other RBCs, leukocytes and platelets, and the vascular lining (Ben-Ami et al., 2003). At low shear stress or upon removal of external forces, RBCs form rouleaux (stacks of RBCs) and three-dimensional aggregates. Aggregate formation affects tissue perfusion and has an impact on hemostasis, probably by affecting the flow behavior of platelets and leukocytes and their interaction with the vascular endothelium (Baskurt and Meiselman, 2013a; Stroobach et al., 2019). Aggregation is likely to be determined for a major part by low-affinity interactions of RBCs with plasma proteins such as fibrinogen and immunoglobulins (Rampling et al., 2004; Weisel and Litvinov, 2019). In addition, changes in aggregation are often accompanied by changes in deformability, and sometimes by changes in cell shape as well (Xue et al., 2013; Li et al., 2014).

## Deformability and Aggregation

Approximately one fifth of the recent papers on RBC deformability also present data on aggregation and *vice versa*, mostly in pathological conditions, but there are few, if any, that try and determine the common factors or mechanisms underlying the observed changes (Xue et al., 2013). In the course of our studies on RBC homeostasis in health and disease, we have accumulated deformability and/or aggregation data of RBCs with various biological backgrounds, such as RBCs of different ages isolated from the circulation of healthy donors (Willekens et al., 2008; Freitas Leal et al., 2019), RBCs of different storage periods in the blood bank (Cluitmans et al., 2012), acanthocytes and otherwise misshapen RBCs from patients with neuroacanthocytosis (De Franceschi et al., 2011; Cluitmans et al., 2015), and RBCs treated with various membrane organization-affecting agents (Dinkla et al., 2012; Cluitmans et al., 2016). In order to shed more light on the mechanisms that underly aging-associated and pathology-related decreases in RBC function, we used the data obtained in these studies to perform a quantitative correlation analysis of the commonly used deformability and aggregation parameters.

It has been shown before that there is a strong linear association between the extent as expressed by the aggregation index (AI) and t_1/2_, the speed by which aggregates form (Baskurt et al., 2009). Analysis of all accumulated data mentioned above confirms this finding, with a Pearson correlation coefficient between AI and t_1/2_ of – 0.87 (Table 1) meaning that if large aggregates are formed, they form faster. There is also a positive correlation between the relaxation time Tr, i.e., the time needed by the RBCs to regain their normal shape from being elongated in the direction of the flow after undergoing a shear rate of 500 s^–1^, and the AI (*r* = 0.43), and between the Tr and the t_1/2_ (*r* = −0.31), indicating that stiffness of RBCs promotes aggregate formation and enhances the speed by which aggregates form. Furthermore, our data show a strong negative correlation (*r* = −0.71) between the calculated maximal deformability EI_max_ and SS_1/2_, the shear stress at which half the EI_max_ is reached (Table 1), demonstrating that elongation is a function of less resistance to deformation. A similar relationship between these deformability parameters has been described for RBCs that had been treated with various concentrations of glutaraldehyde *in vitro* (Baskurt and Meiselman, 2013b; Xue et al., 2013).

**TABLE 1:** Determination of the correlation between deformability and aggregation.

	*r*	*R*^2^	*P* (two-tailed)	*N*
EI_max_ vs. SS_1/2_	–0.7085	0.502	<0.0001	140
AI vs. t_1/2_	–0.8662	0.7504	<0.0001	121
EI_max_ vs. AI	0.2126	0.0452	0.0192	121
EI_max_ vs. AMP	–0.119	0.01415	0.1937	121
EI_max_ vs. t_1/2_	–0.1705	0.02906	0.0616	121
SS_1/2_ vs. AI	–0.2607	0.06794	0.0039	121
SS_1/2_ vs. AMP	0.3482	0.1212	<0.0001	121
SS_1/2_ vs. t_1/2_	0.2511	0.06303	0.0055	121
Tr vs. EI_max_	0.5198	0.2702	<0.0001	121
Tr vs. AI	0.4282	0.1834	<0.0001	121
Tr vs. AMP	–0.4547	0.2067	<0.0001	121
Tr vs. t1/2	–0.3129	0.09791	0.0005	121
Tr vs. SS_1/2_	–0.5874	0.3451	<0.0001	121
Ratio [SS_1/2_/EI_max_] vs. AI	–0.2592	0.06716	0.0041	121
Ratio vs. AMP	0.2647	0.07006	0.0033	121
Ratio vs. t_1/2_	0.2392	0.05723	0.0082	121
Ratio vs. Tr	–0.5918	0.3502	<0.0001	121
Ratio vs. EI_max_	–0.8566	0.7338	<0.0001	140
Ratio vs. SS_1/2_	0.9658	0.9328	<0.0001	140

*RBCs were isolated and their deformability and aggregation characteristics were determined by ektacytometry using a laser-assisted optical rotational cell analyzer (Lorrca Maxis, RR Mechatronics, Hoorn, Netherlands) as described previously (Cluitmans et al., 2012; Da Costa et al., 2016). Deformability was assessed using the elongation index (EI) at various shear stress (0.3 to 30 Pa), yielding the maximal elongation (EI_max_), i.e., the calculated EI at infinite shear stress, and SS_1/2_, i.e., the shear stress at which EI is half of the EI_max_. Aggregation characteristics are assessed using: (1) the shape recovery time Tr, i.e., the time needed by the RBCs to regain their normal shape (Dobbe et al., 2003) after undergoing a shear rate of 500 s^–1^ (shear stress of 1.5 Pa); (2) the aggregation index AI, defined as the decrease in intensity of scattered light during 10 s following disaggregation; (3) the t_1/2_, the rate at which aggregates are formed (Da Costa et al., 2016). Blood was obtained with informed consent and the studies were carried out as described before (De Franceschi et al., 2011; Dinkla et al., 2012; Cluitmans et al., 2015, 2016), in accordance with the CCMO guidelines of the Medical Ethical Committee of the Radboud University Medical Center (file numbers 2007-148, 2013-381, 2018-4421).*

Aggregability is affected by cellular factors such as cell morphology and surface properties, and by the environment. This is illustrated by the effect of RBC age on aggregation characteristics, the variability in RBC aggregability between donors and between species, and by the findings on the correlation between plasma viscosity and aggregation (Rampling et al., 2004; Simmonds et al., 2013). However, the underlying mechanisms are far from clear. Since cell shape and membrane composition are closely related to deformability as well, we analyzed the association between the main deformability and aggregation parameters. The strongest correlation between aggregation and deformability parameters was found between the shape recovery relaxation parameter Tr and the deformability characteristics SS_1/2_ (*r* = −0.59) and EI_max_ (*r* = 0.52), meaning that a rapid recovery from relaxation correlates with a low shear stress required for half maximal elongation and with a strong maximal elongation. Indeed, although Tr is one of the outcome of the aggregation measurement protocol of the Lorrca (RR Mechatronics, Hoorn, Netherlands), Tr is actually a deformability parameter. These findings strengthen the relationship between RBC relaxation and deformation capacity, as observed for RBCs in a microcapillary network-mimicking microfluidics device (Cluitmans et al., 2014). Smaller, but equally statistically significant correlation coefficients were found between AI and SS_1/2_ (*r* = −0.26) and between SS_1/2_ and t_1/2_ (*r* = 0.25; Table 1). This relationship between deformation at a shear stress of 2–3 Pa, which is in the same range as the shear stress that RBCs undergo in microcapillaries (Koutsiaris et al., 2007), and the aggregability is strengthened by the statistically significant correlation (*r* = 0.35) between SS_1/2_ and the extent of aggregation AMP (Table 1).

It has been argued that the deformability parameters SS_1/2_ and EI_max_ as such may not always yield satisfactory, relevant information on deformability properties, especially in the case of large changes in EI_max_. The SS_1/2_/EI_max_ ratio is much less affected by such changes, and may be more suitable when comparing RBCs from various populations or with different clinical backgrounds (Baskurt and Meiselman, 2013b). Applying this ratio, we found statistically significant correlations between deformability and the aggregation parameters AI, AMP, t_1/2_ and Tr, that were higher than using SS_1/2_ and EI_max_ separately (Table 1).

Taken together, our quantitative analyses show that there are a several statistically significant correlations between deformability and aggregation parameters. However, the biological relevance of these correlations remains to be established. Based on the *R*^2^ values, there is only a weak relationship between the deformability and aggregation parameters SS_1/2_ and AMP, i.e., maximally 25 percent of the variance in the shear stress at which half of the maximal deformability is reached, is explained by the variance in the extent of aggregation, and vice versa (Table 1). The relationship between Tr, measured as part of the aggregation analysis, and the deformability parameter SS_1/2_ is considerably stronger (Table 1), supporting the interpretation of the relaxation time Tr as a deformability characteristic.

**FIGURE 1:**
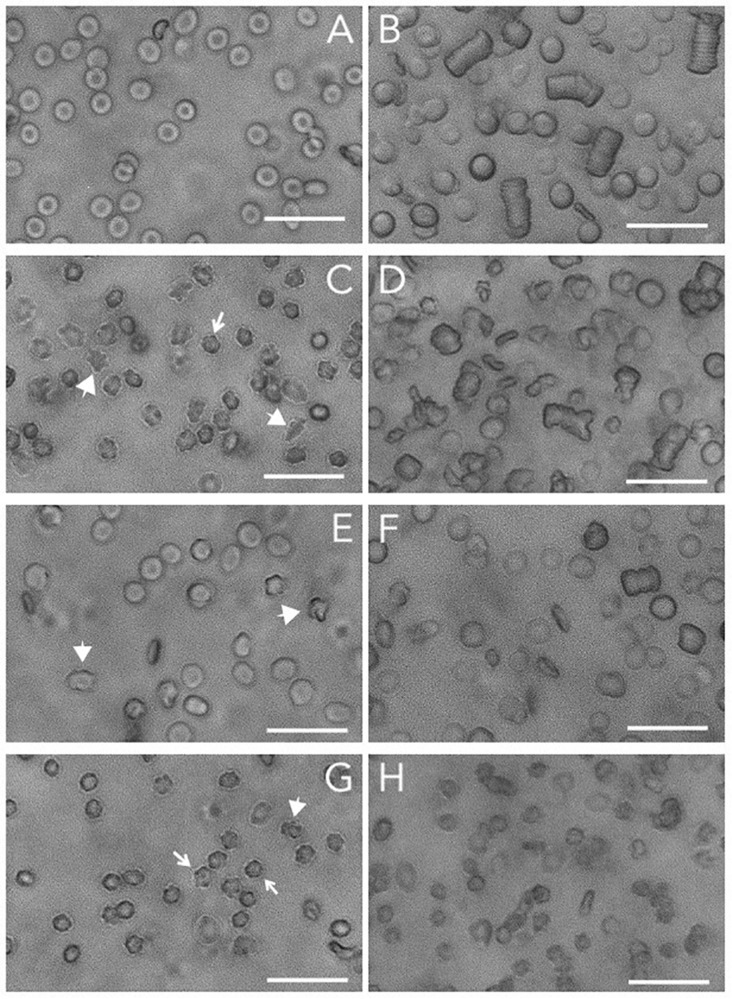
Brightfield microscopy of RBCs and RBC aggregates. Microscopic analyses of RBCs and aggregates were performed by a ZOE Fluorescent Cell Imager (Bio-Rad Laboratories, Hercules, CA, United States). Packed RBCs (1 μL) were resuspended in 199 μL of non-aggregating solution (Ringer) and transferred to an ibidi μ-slide. For aggregation analysis, 2.5 μL of packed RBCs were resuspended in 247.5 μL of plasma. The sample was transferred to an ibidi μ-slide and left for 120 s to allow the RBCs to aggregate. **(A)** control RBCs; **(B)** aggregates of control RBCs; **(C)** RBCs from a neuroacanthocytosis patient; **(D)** RBCs of a neuroacanthocytosis patient after aggregation; **(E,F)**, control RBCs after treatment with orthovanadate before **(E)** and after aggregation **(F)**; **(G,H)** control RBCs after treatment with DIDS before **(G)** and after aggregation **(H)**. The arrows indicate the echinocytes, the large arrowheads point to misshapen RBCs. Treatments were performed as described before (Cluitmans et al., 2012). The length of the bar is 25 μm. Blood was obtained with informed consent and the studies were carried out as described before (De Franceschi et al., 2011; Dinkla et al., 2012; Cluitmans et al., 2015, 2016), in accordance with the CCMO guidelines of the Medical Ethical Committee of the Radboud University Medical Center (file numbers 2007-148, 2013-381, 2018-4421).

It has been speculated that phosphorylation of band 3 is the main molecular determinant controlling deformability and aggregation (Bazanovas et al., 2015). Band 3 plays a key role in many aspects of the RBC aging process, which is associated with alterations in deformability and aggregation (Bosman et al., 2010). Further support for this hypothesis comes from the observation that the formation of acanthocytes and otherwise misshapen RBCs in patients with neuroacanthocytosis is strongly associated with alterations in the Lyn kinase-controlled phosphorylation of membrane proteins including band 3 (De Franceschi et al., 2011, 2014). Thus, to obtain further information on the role of band 3 (phosphorylation) on functional characteristics, we examined the relationship between cell morphology, deformability and aggregability in RBCs from patients with various forms of neuroacanthocytosis (De Franceschi et al., 2011; Cluitmans et al., 2015; Figure 1).

In spite of their aberrant morphology (Figure 1C), acanthocytes deform and relax normally when passing through a microfluidics system that mimics the dimensions (7 μm) and shear stress (2 Pa) of capillaries. However, they are retained when squeezing through a spleen-mimicking device, which is consistent with a decreased deformability (Cluitmans et al., 2015). The negative correlation that was observed between the deformability parameters EI_max_ and SS_1/2_ for control RBCs was observed for neuroacanthocytosis RBCs as well (*r* = −0.59), as was the correlation between the different aggregation parameters AI and t1/2 (*r* = −0.95; Table 2). RBCs from neuroacanthocytosis patients, however, displayed lower Tr values than control RBCs (53 ± 5 vs. 116 ± 7 ms), indicating that these RBCs are more stiff. The neuroacanthocytosis RBCs did not show the correlations between Tr and SS_1/2_ observed for control RBCs (Table 2). Also, there were no statistically significant correlations between the deformability parameters SS_1/2_ and EI_max_ and the aggregation parameters AI and t_1/2_ for the neuroacanthocytosis RBCs (Table 2). In addition, there was no correlation between SS_1/2_ and EI_max_ demonstrating that the intracellular mechanics of these cells is fundamentally different from the one of healthy cells. In contrast, however, a statistically significant correlation was observed between Tr and AMP (the total extent of aggregation), with a correlation coefficient r (0.85) and R^2^ (0.72) that were much higher than those found in control RBCs (Table 2). Overall, these findings suggests that the viscoelastic properties of the lipid bilayer that influence relaxation/deformability become much more pronounced when the band 3-mediated interaction between the membrane and the cytoskeleton is weakened, as in the misshapen RBCs of neuroacanthocytosis patients (De Franceschi et al., 2014).

**TABLE 2:** Determination of the correlations between deformability and aggregation parameters of red blood cells from neuroacanthocytosis patients.

	*r*	*R*^2^	*P* (two-tailed)	*N*
EIi_max_ vs. SS_1/2_	–0.5878	0.3455	0.0444	12
AI vs. t_1/2_	–0.9539	0.9099	<0.0001	10
EImax vs. AI	0.1266	0.01603	0.7274	10
EI_max_ vs. AMP	0.3614	0.1306	0.3048	10
EI_max_ vs. t_1/2_	–0.03048	0.0009291	0.9334	10
SS_1/2_ vs. AI	–0.4562	0.2081	0.1851	10
SS_1/2_ vs. AMP	–0.222	0.04928	0.5376	10
SS_1/2_ vs. t_1/2_	0.4941	0.2442	0.1466	10
Tr vs. EI_max_	0.6559	0.4302	0.0395	10
Tr vs. AI	0.5728	0.3281	0.0835	10
Tr vs. AMP	0.8512	0.7246	0.0018	10
Tr vs. t_1/2_	–0.4576	0.2094	0.1836	10
Tr vs. SS1/2	–0.4139	0.1713	0.2344	10
Ratio [SS1/2/EI_max_] vs. AI	–0.3864	0.1493	0.27	10
Ratio vs. AMP	–0.2781	0.07733	0.4366	10
Ratio vs. t_1/2_	0.3596	0.1293	0.3074	10
Ratio vs. Tr	–0.5511	0.3037	0.0987	10
Ratio vs. EI_max_	–0.8103	0.6566	0.0014	12
Ratio vs. SS_1/2_	0.9449	0.8928	<0.0001	12

*Deformability and aggregation characteristics of the red blood cells from neuroacanthocytosis patients were determined and analyzed as described in the legend to Table 1.*

Microscopy indicates that for the RBCs from neuroacanthocytosis patients the correlation between the kinetics and extent of aggregation is conserved (Figure 1). However, RBCs from neuroacanthocytosis patiens formed more, irregular aggregates than the RBCs from control donors, and these aggregates were smaller and took longer to form (Figure 1). DIDS-induced changes in the organization of band 3 complexes, and orthovanadate-induced, phosphorylation-associated weakening of the connection between the cytoskeleton and the lipid bilayer not only resulted in altered cell morphology, but also in alterations of the shape and size of their aggregates (Figure 1).

## General Discussion and Conclusion

Taken together, the correlations that we found between deformability and aggregation, although weak, suggests that at least one common property is involved in both phenomena. Most of these correlations were absent in the misshapen RBCs of patients with acanthocytosis (Table 1 vs. Table 2). Therefore, the common mechanism is likely to be concentrated around the phosphorylation-controlled binding between the integral membrane protein complexes containing band 3 and the cytoskeleton, that is disturbed in neuroacanthocytosis (De Franceschi et al., 2011, 2014). Data from experimental manipulation of phosphorylation status and from the RBCs of patients with hereditary membranopathies support this hypothesis (Cluitmans et al., 2016; Huisjes et al., 2018). Our present analyses indicate that a disturbance in the association between cytoskeleton and membrane affects deformation more than aggregation, thereby reducing the correlation between both processes. A similar conclusion may be drawn from the apparent lack of correlation between deformability and aggregation, for example in red blood cells of various age (Bosch et al., 1994; Cluitmans et al., 2012; of and of patients with diabetes mellitus and coronary artery disease (Keymel et al., 2011; Preibsch et al., 2017). In addition, our present neuroacanthocytosis data in combination with the data obtained through pharmacological modulation of the band 3 complex, provide analytic approaches for delineating the contribution of various membrane components – and their interactions – to deformability and aggregation.

We emphasize that these conclusions are likely to be influenced by the methods and device that are used to measure deformability, and possibly aggregation as well (Dobbe et al., 2003; Baskurt et al., 2009; Da Costa et al., 2016; Huisjes et al., 2018). For example, we have observed that deformability-assessing methods that employ shear stresses and flow conditions similar to those experienced by RBCs in the capillary system, such as a microfluidics device, yield results that are different from those employing larger forces, such as a bead-sorting device and ektacytometry (Deplaine et al., 2011; Cluitmans et al., 2014, 2015, 2016; Da Costa et al., 2016). In addition, quantitative aggregation measurement by ektacytometry alone is likely to obscure any insight into underlying mechanisms, since the resulting parameters may not always reflect the shape and/or size of the aggregates. One example is the relaxation parameter Tr, which is one of the outcomes of most quantitative aggregation analyses, that shows a higher correlation with deformability than with aggregation parameters (Table 1). Simultaneous microscopic analysis may therefore be valuable in distinguishing between the various factors that affect cell-cell interactions. Microscopy of RBCs from various patients or after treatment reveals, in most cases, considerable heterogeneity in cell size and shape (Cluitmans et al., 2012, 2015, 2016). This heterogeneity may be reflected in variations in the deformability and aggregation parameters, especially in the case of a relatively low number of available patient samples, and may underlie the strong decrease in statistical significance of the correlations between deformability and aggregation for the RBCs observed when comparing the data of control RBCs with those from the neuroacanthocytosis patients (cf. Tables 1, 2). Functional heterogeneity may also be affected by the natural history of the red blood cells and red blood cell fractions, as postulated for the exposure of removal signals and the behavior in a microfluidics device of red blood cells of healthy donors (Cluitmans et al., 2012, 2014; Dinkla et al., 2014), and for the age-associated and disease-related heterogeneity in deformability and aggregation in red blood cells of patients with chronic venous disease (Sloczynska et al., 2015).

In conclusion, our current data show clear and rationally accessible correlations between various deformability and aggregation parameters. These correlations are lost in the RBCs of patients with neuroacanthocytosis, thereby identifying band 3 as a key molecular determinant orchestrating both functions.

## Author Contributions

DL and JF collected the data. DL and GB performed the correlation analyses. DL, JF, RB, and GB wrote the manuscript.

## Conflict of Interest

The authors declare that the research was conducted in the absence of any commercial or financial relationships that could be construed as a potential conflict of interest.
